# Feature tracking cardiovascular magnetic resonance reveals recovery of atrial function after acute myocarditis

**DOI:** 10.1007/s10554-022-02576-1

**Published:** 2022-03-21

**Authors:** J. N. Schneider, C. Jahnke, E. Cavus, C. Chevalier, S. Bohnen, U. K. Radunski, K. A. Riedl, E. Tahir, G. Adam, P. Kirchhof, S. Blankenberg, G. K. Lund, K. Müllerleile

**Affiliations:** 1 Department of Cardiology, University Heart and Vascular Center Hamburg, Martinistrasse 52, 20246 Hamburg, Germany; 2 Department of Cardiology, Asklepios Clinic St. Georg, Hamburg, Germany; 3 Department of Cardiology, Regio Clinics Pinneberg and Elmshorn, Hamburg, Germany; 4 Department of Diagnostic and Interventional Radiology and Nuclear Medicine, University Medical Center Hamburg-Eppendorf, Hamburg, Germany; 5 German Center for Cardiovascular Research (DZHK), Partner Site Hamburg/Kiel/Lübeck, Hamburg, Germany

**Keywords:** CMR-feature-tracking, Atrial strain analysis, Infarct-like myocarditis, Cardiomyopathy-like myocarditis

## Abstract

Follow-up after acute myocarditis is important to detect persisting myocardial dysfunction. However, recovery of atrial function has not been evaluated after acute myocarditis so far. Thirty-five patients with strictly defined acute myocarditis underwent cardiovascular magnetic resonance (CMR, 1.5 T) in the acute stage at baseline (BL) and at 3 months follow-up (FU). The study population included 13 patients with biopsy-proven “cardiomyopathy-like” myocarditis (CLM) and 22 patients with “infarct-like” (ILM) clinical presentation. CMR feature tracking (FT) was performed on conventional cine SSFP sequences. Median LA-GLS increased from 33.2 (14.5; 39.2) at BL to 37.0% (25.2; 44.1, P = 0.0018) at FU in the entire study population. Median LA-GLS also increased from 36.7 (26.5; 42.3) at BL to 41.3% (34.5; 44.8, P = 0.0262) at FU in the ILM subgroup and from 11.3 (6.4; 21.1) at BL to 21.4% (14.2; 30.7, P = 0.0186) at FU in the CLM subgroup. Median RA-GLS significantly increased from BL with 30.8 (22.5; 37.0) to FU with 33.7% (26.8; 45.4, P = 0.0027) in the entire study population. Median RA-GLS also significantly increased from 32.7 (25.8; 41.0) at BL to 35.8% (27.7; 48.0, P = 0.0495) at FU in the ILM subgroup and from 22.8 (13.1; 33.9) at BL to 31.0% (26.0; 40.8, P = 0.0266) at FU in the CLM subgroup. Our findings demonstrate recovery of LA and RA function by CMR-FT strain analyses in patients after acute myocarditis independent from clinical presentation. Monitoring of atrial strain could be an important tool for an individual assessment of healing after acute myocarditis.

## Introduction

Follow up of patients after acute myocarditis is important in order to detect persisting or developing heart failure (HF) as well as enabling a safe return to physical activity [1]. In order to identify patients at risk and optimize treatment, current ESC guidelines recommend follow-up by cardiovascular magnetic resonance (CMR) imaging if the initial CMR showed acute inflammation [2]. Besides inconspicuous troponin and ECG, current guidelines demand normalized left ventricular ejection fraction (LVEF) as prerequisite for return to physical activity/sport [2]. Beyond LVEF, current guidelines advocate global longitudinal strain (GLS) as an additional parameter with incremental value to quantify LV-dysfunction [3]. Recent studies showed the capability of left ventricular global longitudinal strain (LV-GLS) to predict outcome in patients with chronic heart failure (HF) due to ischemic and non-ischemic dilated cardiomyopathy as well as in patients with myocarditis [4–6]. CMR strain analysis was recently applied in acute myocarditis and revealed impaired LV, left atrial (LA) and right ventricular (RV) systolic function [7–11]. In particular, Luetkens et al. recently suggested CMR derived longitudinal LV-strain as new parameter to predict functional recovery after acute myocarditis and further demonstrated improved LV- and RV-strain at the time of follow-up [10].

Besides ventricular function, the relevance of atrial function is increasingly recognized as an important factor in patients with HF: Left atrial (LA) dilatation represents a strong predictive marker in patients with dilatative cardiomyopathy (DCM) [12]. A recent meta-analysis of studies investigating heart failure with preserved ejection fraction (HFpEF) revealed that reduced LA-reservoir strain precedes changes in LVEF [13]. Furthermore, decreased LA reservoir strain was found to be of prognostic value in HFpEF, but also in heart failure with reduced ejection fraction (HFrEF) [13, 14]. The diagnostic value of LA-strain parameters has been evaluated in acute myocarditis before, but there are currently no data on atrial strain after acute myocarditis [8, 9]. Right atrial (RA) function has been neglected so far. However, CMR feature tracking (FT) recently revealed a prognostic role of RA function in HF patients [15]. In patients with acute myocarditis, Dick et al. recently showed a trend towards reduced RA reservoir strain values [8]. In summary, atrial function is of increasingly recognized value in several clinical settings, but little is known on development of atrial function after acute myocarditis. Therefore, this study evaluated CMR-FT derived myocardial strain of all four cardiac chambers in order to assess atrial and ventricular function within the first 3 months after acute myocarditis.

## Methods

### Study population

The local ethics committee approved the study (PV3987) and written informed consent was obtained from all participants. This study is based on additional analyses in a subgroup of 35 consecutive patients of a recently published study population with strictly defined acute/active myocarditis, who underwent CMR at baseline (BL) and at 3 months follow-up (FU). We have published a detailed description of the entire study population before [11]. Briefly, 78 patients with clinically suspected myocarditis were prospectively included. Besides a structured interview, cardiac evaluation included the collection of blood samples as well as the performance of CMR, which was repeated after 3 months at FU. Of the 78 included patients with clinically suspected myocarditis, 48 fulfilled a strict definition of acute myocarditis: Acute myocarditis was defined by endomyocardial biopsy (EMB) in patients with “cardiomyopathy-like” (CLM) presentation and by the combination of recent onset of chest pain, dynamically elevated troponin T values and typical, focal, non-ischemic LGE patterns by CMR in patients who presented with “infarct-like” myocarditis (ILM) [11, 16]. From 39 patients, who underwent 3-months FU, 4 patients had to be excluded from this analysis due to insufficient image quality to perform atrial strain measurements of the left and right atrium thus leaving 35 patients in the final study population for this study [17]. Medical treatment of patients was determined independent from participation in this study in agreement with current recommendations [1]. Thirty patients (86%) were treated with beta-blockers, 28 patients (80%) with angiotensin converting enzyme inhibitors/angiotensin II antagonists, 11 patients (31%) with aldosterone antagonists and 11 patients (31%) with loop diuretics. Two patients (6%) received immunosuppressive therapy guided by endomyocardial biopsy.

### CMR protocol and data analyses

**Fig. 1:**
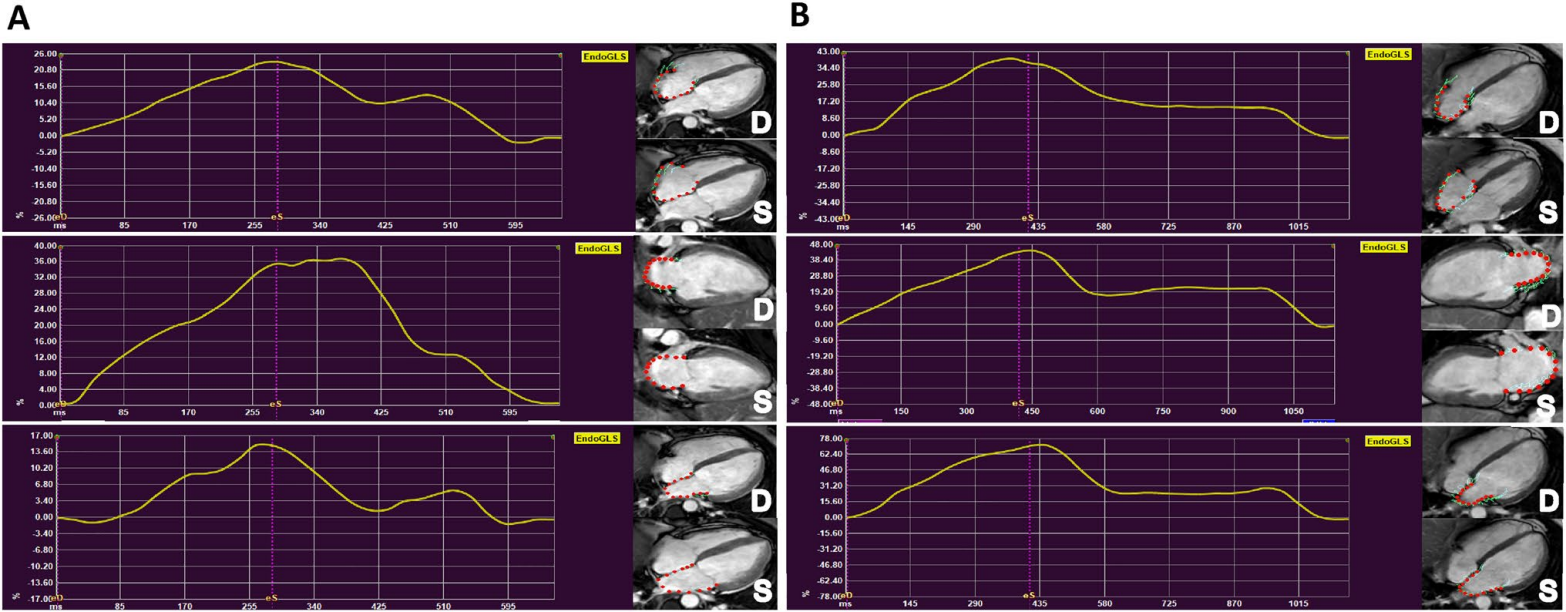
Exemplary strain curves for peak systolic atrial strain. CMR-FT of peak-systolic atrial strain at baseline (**A**) and follow-up (**B**). Strain contours are displayed at end-diastole (D) and end-systole (S). Myocardial deformation is visualized through the red dots following the green lines. In this patient RA-GLS improved from 25.8 at BL to 41.6% at FU. LA-GLS (combined from two-chamber and four-chamber LA-GLS) increased from 26.4 at BL to 56.6% at FU. Please note the different scale of displayed graphs, automatically generated by the software

The CMR protocol was conducted as previously described [11, 18]. Briefly, CMR was performed at 1.5-T (Achieva, Philips Medical Systems, Best, The Netherlands). Besides conventional cine SSFP sequences, the protocol included edema-sensitive, early myocardial enhancement and late gadolinium enhancement (LGE) sequences as well as T1 and T2 mapping [11]. Two patients of our study population had a history of atrial fibrillation, but none of the patients had prevalent atrial fibrillation at the time of CMR acquisition. In addition to our recently published analyses, CMR-FT measurements were performed using dedicated software Medis Suite MR (Medis Medical Imaging, Leiden, The Netherlands) as described before [19]. Myocardial LV-Strain parameters were measured in short- as well as long-axes orientations, employing endocardial as well as epicardial contours according to current standard [20, 21]. RV and RA strain were measured in the 4-chamber view, LA strain in the 2- and 4-chamber view respectively. Biplane left atrial GLS (LA_Bi_GLS) and volumes were calculated as the mean value of two and four-chamber measurements. In the following paragraphs, data referred to as “LA” represent biplane values, if not further specified. LV global myocardial strain was assessed as longitudinal (GLS), circumferential (GCS) as well as radial strain (GRS) employing endo- as well as epicardial contours. GLS for the thin walls of LA, RA and RV was obtained from endocardial contours, since epicardial contours are only available for LV strain by the Medis software (Medis Medical Imaging, Leiden, The Netherlands) (Fig. 1). Presented strain parameters represent peak systolic strain values. The maximum of LA/RA was defined at LV end-systole whereas the minimum was defined at LV end-diastole. Due to the superior reproducibility, reported atrial strain values represent total atrial strain (ε_S_) and therefore describe atrial reservoir function [22].

### Echocardiography

In order to classify diastolic dysfunction for this sub-cohort-study, echocardiographic data, derived during the patients’ hospitalization, was utilized where available. The classification of diastolic dysfunction was performed as recommended by Nagueh et al. [23]. Unfortunately tissue-tracking was not included in the echo protocol.

### Statistical analysis

Statistical analyses were performed using MedCalc for Windows, version 13.3.3.0 (MedCalc Software, Ostend, Belgium). Continuous data are presented as median with first and third quartiles (Q1–Q3). BL and FU parameters were compared using Wilcoxon Test (paired samples) test. Diastolic dysfunction of ILM and CLM was compared by Fisher’s exact test. Twenty randomly selected data sets were analyzed independently by two experienced observers and inter-observer agreement was assessed by calculating intra-class-correlation coefficients (ICC). Two-sided P-values were calculated and P < 0.05 defined as statistically significant. We did not apply a correction for multiple testing. P < 0.05 was defined as statistically significant.

## Results

### Inter-observer agreement of strain measurements

LA-GLS provided an excellent intraclass correlation coefficient (ICC) with 0.943 (95% CI 0.863; 0.977). RA-GLS also offered a very good ICC with 0.878 (95% CI 0.717; 0.950). ICCs were excellent as well for LV-strain analysis with LV_SAX_GCS 0.958 (95% CI 0.564; 0.989) and LV_LAX_GLS 0.922 (95%-CI 0.434; 0.979). Modest ICCs were found for LV-GRS with LV_SAX_GRS 0.575 (0.077; 0.824) and LV_LAX_GRS 0.686 (0.366; 0.862).

### Atrial recovery

Median left atrial ejection fraction (LAEF) significantly increased from BL to FU in the entire study population (49.5% (31.1; 59.0) vs. 59.5% (40.8; 66.5); P = 0.0019) as well as in the subgroups (Tables 1 and 2). Median LA volume index (LAVi), showed a significant decrease from BL to FU in both subgroups with the most profound reduction in CLM patients from 62.5 (51.4; 77.4) at BL to 20.1 ml/m^2^ (18.3; 27.2) at FU (Table 2b). Median LA-GLS increased from 33.2 (14.5; 39.2) at BL to 37.0% (25.2; 44.1, P = 0.0018) at FU in the entire study population. The increase in median LA-GLS remained significant after stratification into both subgroups: Median LA-GLS increased from 36.7 (26.5; 42.3) at BL to 41.3% (34.5; 44.8, P = 0.0262) at FU in ILM patients and from 11.3 (6.4; 21.1) at BL to 21.4% (14.2; 30.7, P = 0.0186) at FU in CLM patients.

**Table 1:** CMR parameters at Baseline (BL) and at 3 months Follow-up (FU)

Parameter	BL	FU	P-value
LVEDVI (ml/m^2^)	83.0 (74.0; 102.0)	78.0 (71.0; 95.5)	**P = 0.0014**
LVESVI (ml/m^2^)	36.0 (28.0; 56.0)	28.0 (22.3; 42.5)	**P < 0.0001**
LVSVI (ml/m^2^)	44.0 (35.3; 51.0)	47.0 (42.0; 54.8)	**P = 0.0043**
LV_LAX_GLS (%)	−16.5 (−18.2; −10.9)	−19.6 (−21.6; −18.2)	**P = 0.0001**
LV_LAX_GRS (%)	45.6 (31.8; 60.4)	59 (45.8; 72.2)	**P = 0.0013**
LV_SAX_GCS (%)	−14.6 (−18.1; −9.7)	−17.2 (−20.2; −13.3)	**P = 0.0010**
LV_SAX_GRS (%)	60.7 (38.7; 70.0)	70.3 (59.5; 92.7)	**P = 0.0002**
RVEF (%)	54.0 (48.3; 59.8)	59.0 (55.0; 61.8)	**P = 0.0059**
RVEDVI (ml/m^2^)	80.1 (72.1; 85.2)	72.00 (62.3; 86.0)	P = 0.0553
RVESVI (ml/m^2^)	35.1 (31.2; 43.8)	30.5 (25.7; 37.0)	**P = 0.0001**
RVSVI (ml/m^2^)	41.9 (35.5; 44.6)	41.6 (34.7; 48.8)	P = 0.2089
RV-GLS (%)	-21.5(−24.2; −17.8)	−22.7 (−24.6; −19.9)	P = 0.1792
LAEF (%)	49.5 (31.1; 59.0)	59.5 (40.8; 66.5)	**P = 0.0019**
LAVI (ml/m^2^)	45.0 (35.2; 58.8)	29.5 (20.5; 33.4)	**P < 0.0001**
LA-GLS (%)	33.2 (14.5; 39.2)	37.0 (25.2; 44.1)	**P = 0.0018**
RAEF (%)	45 (37.3; 54.0)	48 (36.2; 56.0)	P = 0.0913
RAVI (ml/m^2^)	41.8 (34.3; 56.4)	39.9 (32.3; 53.7)	P = 0.3257
RA-GLS (%)	30.8 (22.5; 37.0)	33.7 (26.8; 45.4)	**P = 0.0027**

Values are presented as median with fist (Q1) and third quartiles (Q3)

*P*-values in bold are below the level of significance 0.05

*EDVI* end-diastolic volume index, *EF* ejection fraction, *ESVI* end-systolic volume index, *FU* follow up, *GCS* global circumferential strain, *GLS* global longitudinal strain, *GRS* global radial strain, *LA* left atrium, *LAVI* left atrial volume index, *LAX* long axis, *LV* left ventricle, *RA* right atrium, *RV* right ventricle, *SAX* short axis, *SVI* stroke volume index, “LA- “parameters represent calculated LA-Biplane values form 2- and 4-chamber view

**Table 2:** CMR parameters at BL and at 3 months Follow-up (FU) in patients with infarct-like (a) and cardiomyopathy-like myocarditis (b)

Parameter	BL	FU	P-Value
a			
LVEF%	61.0 (55.0; 66.0)	63.5 (62.0; 72.0)	**P = 0.0015**
LVEDVI (ml/m^2^)	77.0 (69.0; 86.0)	74.5 (71.0; 85.0)	P = 0.1532
LVESVI (ml/m^2^)	30.0 (26.0; 36.0)	26.0 (22.0; 29.0)	**P = 0.0001**
LVSVI (ml/m^2^)	50.0 (41.0; 53.0)	48.5 (45.0; 56.0)	P = 0.2162
LV_LAX_GLS (%)	−17.6 (−19.3; −16.4)	−20.8 (−21.7; −18,9)	**P = 0.0017**
LV_LAX_GRS (%)	56.0 (47.2; 66.7)	67.7 (56.3; 75.0)	**P = 0.0392**
LV_SAX_GCS (%)	−17.54(−19.9; −14.6)	−18.7 (−21.1; −15.7)	**P = 0.0496**
LV_SAX_GRS (%)	64.4 (57.0; 74.0)	76.3 (63.0; 95.2)	**P = 0.0026**
RVEF (%)	57.0 (53.0; 61.0)	59.5 (55.0; 62.0)	P = 0.0918
RVEDVI (ml/m^2^)	77.1 (72.1; 85.4)	77.2 (65.1; 89.4)	P = 0.8076
RVESVI (ml/m^2^)	33.4 (29.8; 38.7)	29.7 (25.7; 37.1)	**P = 0.0208**
RVSVI (ml/m^2^)	43.5 (41.5; 48.0)	48.0 (39.1; 50.4)	P = 0.4549
RV-GLS (%)	−22.7 (−25.1; −21.5)	−22.6 (−24.1; −19.9)	P = 0.2234
LAEF (%)	58.3 (47.5; 61.0)	62.5 (55.0; 67.0)	**P = 0.0273**
LAVI (ml/m^2^)	41.8 (32.3; 47.3)	30.8 (28.1; 33.6)	**P = 0.0022**
LA-GLS (%)	36.7 (26.5; 42.3)	41.3 (34.5; 44.8)	**P = 0.0262**
RAEF (%)	46 (38; 54)	48.5 (37; 57)	P = 0.2024
RAVI (ml/m^2^)	41.5 (34.1; 48.0)	38.5 (32.8; 46.0)	P = 0.4852
RA-GLS (%)	32.7 (25.8; 41.0)	35.8 (27.7; 48.0)	**P = 0.0495**
b			
LVEF%	33.0 (16.8; 43.3)	49.0 (34.8; 65.5)	**P = 0.0007**
LVEDVI (ml/m^2^)	117.0 (85.8; 181.5)	98.0 (72.5; 129.5)	**P = 0.0024**
LVESVI (ml/m^2^)	78.0 (50.0; 132.5)	52.0 (29.5; 84.8)	**P = 0.0017**
LVSVI (ml/m^2^)	35.0 (26.8; 41.0)	43.0 (38.5; 52.5)	**P = 0.0017**
LV_LAX_GLS (%)	−10.4 (−13.3; −5.8)	−13.0 (−19.8; −9.8)	**P = 0.0093**
LV_LAX_GRS (%)	27.9 (20.9; 33.9)	42.1 (23.8; 62.5)	**P = 0.0068**
LV_SAX_GCS (%)	−8.9 (−11.8; −5.4)	−12.0 (−17.5; −7.1)	**P = 0.0017**
LV_SAX_GRS (%)	33.7 (20.4; 39.5)	54.7 (34.5; 73.2)	**P = 0.0171**
RVEF (%)	48.0 (33.8; 55.0)	57.0 (47.8; 59.5)	**P = 0.0398**
RVEDVI (ml/m^2^)	84.2 (73.0; 88.4)	69.0 (61.7; 74.4)	**P = 0.0171**
RVESVI (ml/m^2^)	45.7 (34.6; 58.13)	31.1 (25.9; 36.8)	**P = 0.0002**
RVSVI (ml/m^2^)	33.2 (29.9; 41.9)	34.7 (33.7; 43.5)	P = 0.3054
RV-GLS (%)	−17.8 (−18.8; −11.2)	−23.5 (−24.7; −14.2)	P = 0.3396
LAEF (%)	30.5 (16.0; 48.6)	38.0 (32.5; 50.2)	**P = 0.0327**
LAVI (ml/m^2^)	62.5 (51.4; 77.4)	20.1 (18.3; 27.2)	**P = 0.0007**
LA-GLS (%)	11.3 (6.4; 21.1)	21.4 (14.2; 30.7)	**P = 0.0186**
RAEF (%)	42 (30.8; 55.3)	45 (34.5; 52.5)	P = 0.3054
RAVI (ml/m^2^)	53.1 (33.6; 62.3)	43.0 (31.7; 58.4)	P = 0.4143
RA-GLS (%)	22.8 (13.1; 33.9)	31.0 (26.0; 40.8)	**P = 0.0266**

Values are presented as median with fist (Q1) and third quartiles (Q3)

*P*-values in bold are below the level of significance 0.05

*EDVI* end-diastolic volume index, *EF* ejection fraction, *ESVI* end-systolic volume index, *FU* follow up, *GCS* global circumferential strain, *GLS* global longitudinal strain, *GRS* global radial strain, *LA* left atrium, *LAVI* left atrial volume index, *LAX* long axis, *LV* left ventricle, *RA* right atrium, *RV* right ventricle, *SAX* short axis, *SVI* stroke volume index; “LA- “parameters represent calculated LA-Biplane values form 2- and 4chamber view

**Fig. 2:**
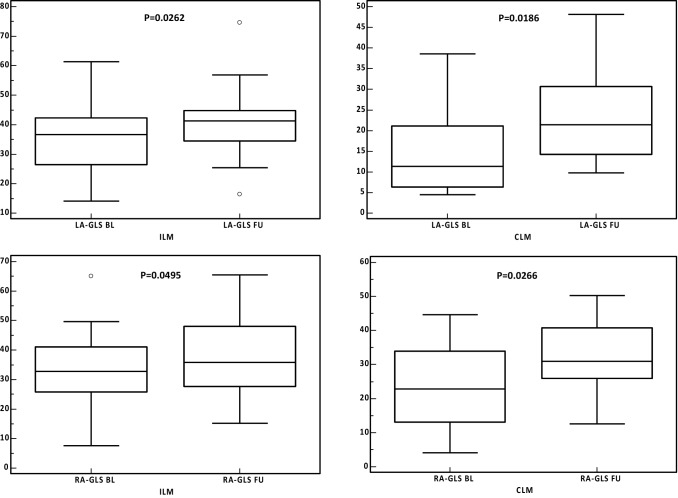
Atrial strain at baseline (BL) and follow-up (FU). Left atrial (LA) and right atrial (RA) strain at baseline (BL) and at follow up (FU). Box-Whisker plots of median left atrial global longitudinal strain (LA-GLS) and right atrial global longitudinal strain (RA-GLS) at BL and FU for infarct-like myocarditis (ILM) and cardiomyopathy-like myocarditis (CLM)

There was no significant change in median right atrial ejection fraction (RAEF) and median right atrial volume index (RAVi) from BL to FU, neither in the entire study population, nor in the subgroup analyses (Tables 1 and 2). However, median RA-GLS significantly increased from BL with 30.8 (22.5; 37.0) to FU with 33.7% (26.8; 45.4, P = 0.0027) in the entire study population. This finding remained consistent in both subgroups: RA-GLS increased from 32.7 (25.8; 41.0) to 35.8% (27.7; 48.0, P = 0.0495) in ILM patients and from 22.8 (13.1; 33.9) to 31.0% (26.0; 40.8) in CLM patients (P = 0.0266), (Table 2, Fig. 2).

There was a significant, inverse correlation of changes in heart rate between BL and FU with changes in LA-GLS (− 0.515 (− 0.0803; 0.630) P = 0.0022) and RA-GLS (− 0.351 (− 0.613; − 0.0201) P = 0.0387).

### Ventricular recovery

Median LVEF significantly improved in the entire study population (Table 1). In CLM patients, median LVEF significantly improved from 33 (16.8; 43.3) at BL to 49% (34.8; 65.5, P = 0.0007) at FU. In ILM patients, median LVEF increased from 61 (55; 66) at BL to 64% (62; 72) at FU (P = 0.0015). Median left ventricular end-diastolic volume index (LVEDVi) significantly decreased from BL to FU in CLM and ILM (Table 2). LV-strain showed a significant increase from BL to FU in the entire study population for all parameters (Table 1): Median LV_LAX_GLS increased from BL to FU in CLM with − 10.4% (− 13.3; − 5.8) vs. − 13.0% (− 19.8; − 9.8, P = 0.0093), but also in ILM with − 17.6% (− 19.3; − 16.4) vs. − 20.8% (− 21.17; − 18.9, P = 0.0017) (Table 2). Median LV_SAX_GCS also significantly increased from BL to FU in both subgroups (Table 2). Sufficient data to classify LV diastolic dysfunction (DD) by echocardiography were available in 25 patients. All 25 patients had DD at BL. Eighteen (72%) patients were classified as DD I° and 7 (28%) as DD ≥ II°. At baseline, severe DD ≥ II° was more frequent in CLM patients (n = 6, 60%) compared to ILM patients (n = 1, 7%, P = 0.0068). There was a significant decrease in E/e’ from 8.3 (5.4; 12.1) at BL to 5.9 (5.1; 8.1, P = 0.0046) at FU.

Median right ventricular ejection fraction (RVEF) significantly improved from 48.0 (33.8; 55.0) at BL to 57.0% (47.8; 59.5, P = 0.0398) at FU in CML, but not in ILM (Table 2). Median right ventricular end-diastolic volume index (RVEDVi) significantly decreased from BL to FU in both subgroups. There were no significant differences in median RV-global longitudinal strain (RV-GLS) between BL with − 21.5% (− 24.2; − 17.8) and FU with − 22.7% (− 24.6; − 19.9, P = 0.1792) in the entire study population, as well as in the subgroup analyses (Tables 1 and 2).

## Discussion

This study evaluated atrial and ventricular function after acute myocarditis by CMR-FT. To the best of our knowledge, development atrial function by FT after acute myocarditis has not been investigated so far. Because of its superior reproducibility, we report total atrial strain (ε_S_) as parameter for atrial reservoir function [22]. Our major findings were: First, there was a significant increase in median LA und RA strain from BL to FU. Second, we found a significant increase in LV, but not in RV strain from BL to FU. Third, atrial and LV functional improvement was found in patients with “cardiomyopathy-like” as well as in patients with “infarct-like” clinical presentation.

### Left atrial recovery

In our analyses, median LA-GLS significantly increased from BL to FU (Table 1, Fig. 2). This finding could be related to two different aspects of myocardial recovery after acute myocarditis: First, receding inflammation of atrial myocardium resulted in recovery of myocardial function in analogy to LV myocardial recovery. However, only few reports have actually provided histological data on atrial involvement in myocardial inflammation [24]. Moreover, atrial tissue characterization by CMR is difficult and requires high resolution sequences [25]. Interestingly, there was a modest to moderate inverse correlation of decrease in heart rate with the increase in LA-strain from BL to FU, which certainly reflects myocardial healing and recovery of cardiac function, but could also constitute a potential confounder.[26]. Second, receding myocardial inflammation on the ventricular level [11] resulted in improved systolic and diastolic function with subsequent decline in LA load [27]. LA-strain was recently found to be associated with the extent of DD in patients with preserved LVEF and to be a sensitive marker for severe DD irrespective of LV systolic function [28, 29]. Furthermore, LA-strain appears to be superior compared to LAVi and E/e’-ratio in classifying DD [29]. In our analysis, CLM patients had a significantly lower median BL LA-GLS with 11.3% (6.4; 21.1) compared to ILM patients with 36.7% (26.5; 42.3). Correspondingly, LV diastolic dysfunction at BL was more pronounced in CLM patients compared to ILM patients. At FU, LAVi and E/E’ showed a significant reduction in the overall study population, indicating an improved diastolic function with cessation of myocardial inflammation. Accordingly, we found a significant increase in overall LA-strain at FU (Table 1). In summary, CMR derived LA-strain seems to reflect improvement of systolic and diastolic LV dysfunction after acute myocarditis [30].

However, recent data indicate an independent, incremental value of LA-strain beyond LV function, e.g. as an early indicator of risk for the development of HF, occurrence of atrial fibrillation, but also stroke risk [31–33]. Of note, LA-strain offers additional information in HFpEF patients [28, 29], but also HFrEF patients, indicating a role for LA-strain measurements in HF independent from LVEF [13, 14]. In particular, Deferm et al. recently demonstrated a prognostic role of LA reservoir strain in patients with acute HFrEF, independent from changes in LA volumes and LV-function [34]. Accordingly, there could be a role for LA-strain measurements to guide therapy in acute HF beyond conventional parameters such as LVEF [35]. In analogy to these findings in other settings, monitoring LA-strain could improve the individual assessment of healing in patients after acute myocarditis, which is one of the major challenges in clinical routine [2, 16]. Nevertheless, larger studies with long-term follow-up are required before clinical implementation of LA-strain measurements.

### Right atrial recovery

We were able to demonstrate a small but significant increase in RA-peak-systolic-GLS from BL to FU in patients with ILM and CLM (Table 2). Analogous to LA-strain, this finding could be related to recovery of atrial myocardial function, but also to improved RV diastolic and systolic function. In patients with acute myocarditis, Dick et al. were able to show a trend towards reduced RA reservoir strain values before [8]. Recently, right heart dysfunction has been found to be of particular relevance in patients with HFpEF, contributing to poor outcome [36]. Jain et al. described significantly reduced right atrial conduit and reservoir strain in patients with HFpEF as well as HFrEF. RA conduit and reservoir strain were found to be independent predictors of mortality irrespective of LVEF or HF status [15]. Tough these studies did not investigate patients with myocarditis in particular, patients with ILM and CLM experience acute HF and can be attributed to HFpEF or HFrEF categories. These findings underline the potential value of monitoring right atrial function in patients after acute myocarditis. Whether RA function in myocarditis provides incremental prognostic information and whether RA strain is able to assess RV diastolic function, needs to be investigated in future studies.

### Left ventricular recovery

Recovery of RV- and LV- strain in patients with acute myocarditis has been described before [10]. Luetkens et al. suggested LV-GLS as a predictor of functional recovery in myocarditis [10]. We were able to confirm an increase of global LV-strain parameters from BL to FU in our data (Table 1). Furthermore, LV_LAX_GLS and LV_SAX_GCS improved from BL to FU independent of clinical presentation (Table 2). The absolute increase of LV_SAX_GCS however was smaller compared with LV_LAX_GLS in the overall study population and just barely significant in ILM patients (Table 2). In CLM, absolute LV_SAX_GCS at baseline was much lower than in ILM patients, mirroring reduced LVEF and increased LV dilatation (Table 2). The results agree with previous findings that showed a more pronounced deterioration of LV_LAX_GLS per reduction of LVEF in percent compared to LV_SAX_GCS [37]. Especially in patients with preserved LVEF, LV_LAX_GLS therefore probably offers a more sensitive description of myocardial contractility compared to LV_SAX_GCS and LVEF, making LV_LAX_GLS the optimal parameter to describe subclinical changes in systolic function [37]. This explains the discrepancy between absolute change of LV_LAX_GLS and LV_SAX_GCS in ILM, where LVEF at baseline was preserved (Table 2a). Furthermore, CMR feature-tracking derived myocardial LV-GLS has been independently associated with mortality in patients with dilated cardiomyopathy [5]. A recent meta-analysis indicated a superior prognostic value of LV-GLS over LVEF in predicting major adverse cardiac events [38]. In particular in patients with acute myocarditis, LV-GLS was superior compared to clinical features, LVEF and LGE in this context [6]. It should therefore be considered to adopt LV strain analyses into routine follow-up of patients after acute myocarditis.

### Right ventricular recovery

Reduced RV function has been described as independent predictor of adverse outcomes [39]. In our cohort, global RV-GLS did not change significantly between BL and FU. Up to now, conflicting data on RV strain have been published: In contrast to our findings, Luetkens et al. reported an increase in RV-GLS from BL to FU in a similar study population [10]. Baessler et al. reported a paradoxically increased, “supernormal” basal RV-strain-rate in patients with acute myocarditis, whereas no change was found in global RV-strain-rate [7]. These divergent findings could be related to different study populations with different degrees of RV involvement, but also to different extent of LV impairment with subsequently increased “secondary” RV load. Further studies are necessary to better understand the role of RV-GLS changes after acute myocarditis.

### Clinical presentation: ILM vs. CLM

Depending on clinical presentation, patients with acute myocarditis can be divided into two major groups: Briefly, patients with chest pain, elevated Troponin levels and ST-alterations on ECG can be classified as having “infarct-like myocarditis” (ILM), whereas patients presenting with symptoms of new-onset heart-failure can be categorized as having “cardiomyopathy-like myocarditis” (CLM) [1, 40, 41]. Despite preserved LVEF, major adverse cardiac events such as recurrent myocarditis, sustained ventricular tachycardia and sudden cardiac death have been attributed to an infarct-like pattern in patients with acute myocarditis [42]. Assessment of cardiac function at baseline and during follow up is routinely performed by echocardiography which offers LVEF as established parameter in characterizing systolic function [1]. Since LVEF can be near normal or preserved in the acute stage of ILM (Table 2a, [41]) monitoring of ejection fraction alone often does not allow for sufficient estimation of cardiac function in all patients. CMR is able to provide complementary tools in this context: While T1 and T2 mapping CMR are able to monitor myocardial inflammation, strain analysis allows the monitoring of myocardial function beyond LVEF [37]. In our cohort, the change in T1 and T2 mapping parameters did not correlate significantly with the observed changes in strain or LVEF. We could observe a significant increase in median LV-GLS from BL to FU in both subgroups, indicating functional improvement independent from LVEF. As mentioned above, LV_LAX_GLS allows for a better description of subclinical changes in contractile dysfunction than LVEF and LV_SAX_GCS, especially in patients with ILM [37]. LV_LAX_GLS therefore constitutes an important parameter depicting LV-systolic dysfunction independent of clinical phenotype in myocarditis. Moreover, the increase in LA-GLS from BL to FU in both subgroups suggests improvement of diastolic function with subsequent relief of LA load beyond recovery of atrial myocardium itself [28, 29]. Upon this premise, CMR-FT seems to offer a phenotype-independent option to assess myocardial function in healing myocarditis [2]. Our group recently published normal values for atrial strain values using the same method and software (Medis Medical Imaging, Leiden, The Netherlands) [19]. In this publication, median two/four chamber LA peak-systolic global longitudinal strain (GLS) were 38.2 (33.0; 43.7)/33.4% (28.4–37.3) and 29.8% (24.1–35.1) for RA-GLS in the control group. Briefly, our findings indicate normalization of median LA- (37.0% (25.2; 44.1)) and RA-GLS (33.7% (26.8; 45.4)) at FU for the overall study population. (Table 1). However, this observation was primarily driven by ILM patients: In ILM patients, we observed an increase of median LA-GLS from (36.7% (26.5; 42.3)) at BL to (41.3% (34.5; 44.8)) at FU (Table 2a). In contrast, CLM patients had lower median LA- (11.3% (6.4; 21.1)) and RA-GLS (22.8% (13.1; 33.9)) values at BL, which improved, but did not fully normalize at FU (LA-GLS 21.4% (14.2; 30.7); RA-GLS 31.0% (26.0; 40.8)) (Table 2b). In line with these findings, strain values of all four cardiac chambers and LVEF remained reduced in CLM patients at FU. Though healing improves cardiac strain also in patients with CML compared to the acute phase, patients need close clinical follow up in order to offer early medical therapy according to current guidelines. [2]

### Limitations

First, our study population was relatively small. Furthermore, the study population is affected by an inherent selection bias, since patients with clinical worsening and subsequent ICD-implantation or heart transplantation were not able to participate at FU. However, our study population is representative for the majority of patients after acute myocarditis, who undergo regular follow-up visits in outpatient settings. Nevertheless, larger studies with long-term follow-up are warranted to better understand the relevance of functional recovery by atrial CMR-FT compared to myocardial tissue characterization, but also conventional biomarkers after acute myocarditis. We observed a significant correlation between changes in LA- and RA-strain with changes in heart rate from BL to FU, which could be explained by myocardial healing. However, changes in heart rate could constitute a potential technical confounder for the observed changes in LA-strain [26]. In addition, though we could demonstrate a very good inter-observer-agreement for the measured strain values, test–retest (inter-scan) variability was not addressed in this study [43].

## Conclusion

Our findings demonstrate recovery of LA and RA function by CMR-FT strain analyses in patients after acute myocarditis independent from clinical presentation. Monitoring atrial strain could be an important tool for an individual assessment of healing after acute myocarditis.

## Abbreviations

BL: Baseline
CI: Confident intervals
CLM: Cardiomyopathy-like myocarditis
CMR: Cardiovascular magnetic resonance
DCM: Dilatative cardiomyopathy
DD: Diastolic dysfunction
EMB: Endomyocardial biopsy
FT: Feature tracking
FU: Follow-up
GCS: Global circumferential strain
GLS: Global longitudinal strain
GRS: Global radial strain
HF: Heart failure
HFpEF: Heart failure with preserved ejection fraction
HFrEF: Heart failure with reduced ejection fraction
ICC: Intra-class-correlation coefficient
ILM: Infarct-like myocarditis
LA: Left atrium
LAEF: Left atrial ejection fraction
LAVI: Left atrial volume index
LAX: Long axis
LV: Left ventricle
LVEDVI: Left ventricular end-diastolic volume index
LVEF: Left ventricular ejection fraction
LVESVI: Left ventricular end-systolic volume index
LVSVI: Left ventricular stroke volume index
Q1: First quartile
RA: Right atrium
RAEF: Right atrial ejection fraction
RAVI: Right atrial volume index
Rho: Spearman’s coefficient of rank correlation
RV: Right ventricle
RVEDVI: Right ventricular end-diastolic volume index
RVEF: Right ventricular ejection fraction
RVESVI: Right ventricular end-systolic volume index
RVSVI: Right ventricular stroke volume index
SAX: Short axis
